# Reasons for difficulties in isolating the causative organism during food-borne outbreak investigations using STEC as a model pathogen: a systematic review, 2000 to 2019

**DOI:** 10.2807/1560-7917.ES.2024.29.49.2400193

**Published:** 2024-12-05

**Authors:** Christina Anthony, Karen Pearson, Rebecca Callaby, Lesley Allison, Claire Jenkins, Alison Smith-Palmer, Marianne James

**Affiliations:** 1 Food Standards Scotland, Aberdeen, United Kingdom; 2 Scottish *E. coli* O157/STEC Reference Laboratory, Edinburgh, United Kingdom; 3 UK Health Security Agency, London, United Kingdom; 4 Public Health Scotland, Glasgow, United Kingdom

**Keywords:** Shiga toxin-producing Escherichia coli, STEC, Foodborne, Outbreak, Investigation, Isolation

## Abstract

**Introduction:**

Food-borne disease outbreak investigations use epidemiological, microbiological and food chain evidence to identify the implicated food and inform risk management actions.

**Aims:**

We used Shiga toxin-producing *Escherichia coli* (STEC) as a model pathogen to investigate the success of outbreak strain isolation from food or environmental samples during outbreak investigations, and examined the factors influencing the chance of isolation.

**Methods:**

We searched for reports of food-borne STEC outbreak investigations worldwide in peer-reviewed and grey literature in line with the Preferred Reporting Items for Systematic Reviews and Meta-Analyses (PRISMA) guidelines.

**Results:**

We found a total of 223 outbreaks suitable for inclusion. Food and/or environmental samples were available for testing in 137 investigations, and the outbreak strain was isolated in 94 (42%) of investigations. We found no significant effect of STEC serovar or size of outbreak on likelihood of successful outbreak strain isolation. Isolation success ranged across different implicated commodities from 86% for beef-related outbreaks to 50% for salads and leafy greens. In 20% of outbreaks with samples available for testing, an additional STEC strain was isolated alongside the outbreak strain and in 6.6%, only an alternative STEC strain was isolated. Risk management action was taken on epidemiological evidence alone in 21 incidents.

**Conclusion:**

The principal reasons why the outbreak strain was not isolated were lack of sample availability and methodological issues concerned with laboratory isolation. We recommend strategies that could improve the likelihood of isolation including the rapid collection of samples based on epidemiological intelligence.

## Introduction

Food-borne illnesses are a major public health concern and a considerable economic burden worldwide. The World Health Organisation (WHO) states that unsafe food causes an estimated 600 million cases of food-borne diseases and 420,000 deaths worldwide each year, with 30% of deaths from food-borne disease occurring among children under 5 years of age [1]. When an outbreak of food-borne disease occurs, epidemiological, microbiological and environmental intelligence are used as part of the outbreak investigation to identify the food vehicle(s) involved, the source of the pathogen and any other contributing factors [2]. Isolating the outbreak strain (as identified from the clinical samples of the outbreak cases) from food or environmental samples is considered strong evidence of a causal link. As well as informing control measures for the current outbreak, being able to attribute an outbreak to a specific food helps contributes towards the development of public health policy on food safety [3].

Shiga toxin-producing *Escherichia coli* (STEC) are a group of Gram-negative bacterial pathogens that exist as normal microbiota (commensal organisms) in ruminant animals such as cattle and sheep but can cause disease in humans. Over 100 different STEC serotypes are associated with human illness [4,5]. Globally, STEC is responsible for around 2,801,000 acute infections, 3,890 cases of haemolytic uraemic syndrome (HUS) and 230 deaths every year [6]. Historically, food-borne STEC outbreaks were most commonly attributed to red meat or meat products or to milk (either raw milk or pasteurisation failure) and milk products [7]. More recently, STEC outbreaks caused by the consumption of fresh produce contaminated with animal faeces have been identified [8]. However, it is recognised that for many STEC outbreak investigations, the outbreak strain is not isolated from the food or environmental source implicated by epidemiological investigations.

The complex nature of epidemiological investigations means that they are often not fully understood by the general public, the media [9] or key stakeholders in the food industry such as food business operators, suppliers and retailers. As a result, the scientific value given to epidemiological evidence in the absence of microbiological evidence is sometimes called into question by these stakeholder groups.

In this report, we use STEC as a model pathogen to investigate the proportion of food-borne outbreaks where the outbreak strain was not isolated from food or environmental samples during the investigation. We chose STEC as a model pathogen due to (i) the availability of a sufficient number of published outbreak investigations, (ii) the public health significance of STEC due to the potential for severe disease and (iii) its relatively short incubation period compared with some other food-borne pathogens which increases the chance of accurate patient recall. Specifically, we identified global food-borne STEC outbreaks reported from January 2000 to August 2019, reviewed how many of these outbreak investigations were successful in isolating the outbreak strain from the implicated food item and/or food production chain, and examined the factors which influenced the success of isolation.

## Method

### Search strategy

We searched for reports of food-borne STEC outbreak investigations worldwide in both peer-reviewed literature and grey (i.e. non-peer reviewed) publications. The following sources were included: Ovid, PubMed, Google Scholar, the United States (US) Centers for Disease Control and Prevention’s (CDC) OutbreakNet Food-borne Outbreak Online Database, Public Health Canada’s public health notices, European Centre for Disease Prevention and Control (ECDC) surveillance and updates on E. coli webpages, the World Health Organisation archives, Australian Government Department of Health reports, the US outbreak database and Health Protection Scotland (HPS) ObSurv Surveillance data reports.

To ensure reports were not missed due to differences in terminology or alternative spellings, the following search terms (from title or abstract) were included in multiple combinations: “Escherichia coli”, “e.coli”, “e. coli”, “e .coli”; “shiga-toxigenic”, “shiga toxigenic”, “shiga-toxin”, “shiga toxin”, “shiga toxin-producing”; “vero cytotoxin”, “vero-cytotoxin”, “verotoxin”, “vero cytotoxin-producing”, “verotoxin-producing”; “enterohemorrhagic”, “enterohaemorrhagic”; “O157”, “O104”, “O111”, “O26”, “O45”; “STEC”, “EHEC”, “VTEC”; “Foodborne”, “food borne”, “foodborne disease$”, “food vehicle”, “Outbreak$”.

We carried out searches of outbreaks with a start date from 1 January 2000 until 31 August 2019. The literature search phase of the review was carried out in September 2019. We recognise that laboratory methods have evolved greatly over the past 20 years, with the addition of ever more specific typing methods and these developments will have been implemented in different countries at different times, so there isn’t a single step change in methodologies to define a date. The year 2000 was chosen as a pragmatic approach that captures the laboratory developments in supporting outbreak investigations over this time. We reviewed the bibliographies of suitable resources to identify any further relevant resources that may not have been captured by the initial search. The search was conducted in line with the Preferred Reporting Items for Systematic Reviews and Meta-Analyses (PRISMA) guidelines and confirming to the PRISMA statement [10].

### Study selection

We used the title and abstract to review each report’s suitability for inclusion in the study, with any study meeting any of the following criteria excluded from further analysis: (i) outbreaks occurring pre-2000, (ii) non-food-borne outbreaks, (iii) full text article not available in English and (iv) outbreaks of types of *E. coli* other than STEC.

We examined each report’s full text and extracted and recorded the following information in Microsoft Excel: year, location, food vehicle (suspected or confirmed), food category, number of cases, STEC serogroups (*E. coli* O157 or non-O157), investigations undertaken, outbreak setting, successful isolation of outbreak strain (yes/no, from food and/or environmental sample), authors’ interpretation of reason for the failure to isolate the outbreak strain (where appropriate), risk management measures taken (where the outbreak strain was not isolated from food or environmental samples), and whether the report was published in a peer-reviewed journal or grey literature. Where it was not mentioned in the investigation report that food and/or environmental samples were taken, we assumed that they were not.

### Data analysis

Author CA, checked by MJ, assigned a critical appraisal score to the data from each report. We considered existing tools for evaluation and critical appraisal of resources, such as the Critical Appraisal Skills Programme (CASP) checklist (http://www.casp-uk.net/), but deemed them unsuitable for use in this review, so developed a novel approach to appraise the data available for each study. A comprehensive food-borne outbreak investigation was deemed to include each of the five key stages: descriptive epidemiology, analytical epidemiology, food investigations, environmental investigations and trace-back investigations. A scoring system was applied so that one point was assigned for each of the five aspects of an outbreak investigation that was mentioned within the resource of interest, resulting in a maximum score of five points.

We used chi-square tests to test the association between outbreak strain isolation success and STEC serotype, outbreak size and availability of samples to test and successful isolation of the outbreak strain from food or environmental samples and outbreak size. In addition, we used a Cochran–Armitage trend analysis to examine if samples are more likely to be taken for certain sizes of outbreak. A generalised linear regression model fitted with a binomial distribution was used to look at the success of isolation of the outbreak strain from different food types in comparison to beef. Beef was used as a comparison as it was the most common food vehicle for isolation of STECs.

Analysis was undertaken using R version 4.2.2 (R Core Team, 2022).

## Results

**Figure 1:**
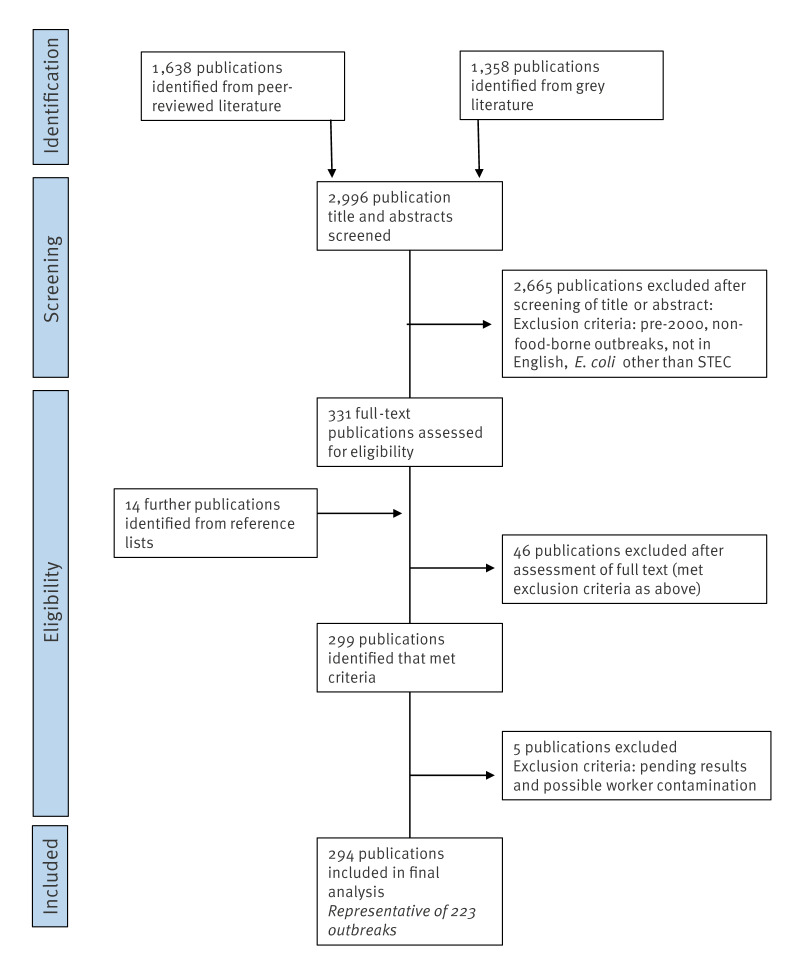
Flow diagram of search strategy used to identify publications, and subsequent outbreaks suitable for inclusion in the systematic review, food-borne outbreak investigations involving STEC (n = 294 publications)

We found a total of 223 outbreaks suitable for inclusion for further analysis following removal of reports which met the exclusion criteria (Figure 1). Where more than one publication was identified for the same outbreak, the information compiled into the spreadsheet was taken from all the publications related to that outbreak.

STEC: Shiga toxin-producing *Escherichia coli*.

Of the 223 STEC outbreaks identified in this systematic review, the majority (n = 184; 83%) were caused by *E. coli* O157. Outbreak case totals ranged from two to 3,842, and the review covered a total of 13,003 cases of illness and 2,664 reported hospitalisations (noting that 60 of the 223 outbreaks did not report the total number of cases hospitalised).

We identified outbreaks from 17 countries for inclusion in the review. The highest number of outbreaks were from the United States (n = 136), the United Kingdom (n = 23), Canada (n = 19), France (n = 7), Germany (n = 4) and Japan (n = 4). Other countries with one or two outbreaks recorded were: Belgium, Denmark, Finland, Iceland, Italy, Netherlands, Norway, Romania, South Korea, Spain and Sweden. Nine outbreaks were recorded as multi-country including the countries named above, and one outbreak including a total of 14 European countries.

**Figure 2:**
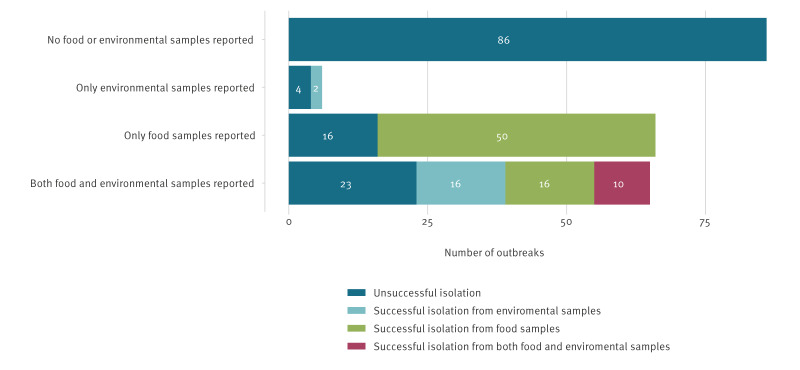
Food-borne STEC outbreak investigations by isolation status and sample source (n = 223 outbreaks)

The outbreak strain was isolated either from food samples, environmental samples or both in 94 (42%) of the 223 outbreaks identified in this review (Figure 2). Outbreak investigations were unable to isolate the outbreak strain from food or environmental samples in the remaining 129 outbreaks (58%), although for 86 of these (67% of outbreaks where the outbreak strain was not detected), no samples were available for testing. 

STEC: Shiga toxin-producing *Escherichia coli*.

For 65 outbreaks, both food and environmental samples were available for testing, with the outbreak strain being isolated in 42 of these (Figure 2). For 10 of these outbreak investigations the outbreak strain was isolated from both food and environmental samples, for 16 the outbreak strain was only detected from the environmental sample, and for the remaining 16 investigations the outbreak strain was only isolated from the food sample. For the 28 outbreaks where environmental samples tested positive for the outbreak strain, the majority (n = 22) came from outdoor settings (animal faeces, irrigation water or swabs from farm settings). The remaining six positive environmental samples were from food handling facilities, including two swabs from infected food handlers.

There was no difference in outbreak strain isolation success between *E. coli* O157 and non-O157 (χ^2^ = 0.4321, df = 1, p = 0.511). The one outbreak where multiple STEC strains were isolated was not included in this chi-square test.

Table 1 shows the isolation status and availability of samples for testing by outbreak size, with outbreak size based on the confirmed case number according to the case definition within the publication. Sample availability was associated with the size of the outbreak (χ^2^ = 15.148, df = 4, p = 0.0044); larger outbreaks were more likely to have samples taken (Cochran-Armitage trend test, z = 9.88, df = 1, p = 0.0017). Isolation success was not associated with the size of the outbreak (χ^2^ = 5.4956, df = 4, p = 0.2401). In addition, using a cut-off outbreak size of ≤ 100 or > 100 people, there was no difference in outbreak size on isolation success (χ^2^ = 1.5374, df = 1, p = 0.215).

**Table 1:** Isolation status and availability of samples for testing by outbreak size, food-borne outbreaks of STEC (n = 223 outbreaks)

Outbreak size	Samples available^a^	Samples not available^b^	Total	Successful isolation^c^	Unsuccessful isolation^d^
	n	n		n	n
< 5	11	11	22	9	2
5–15	36	41	77	25	11
16–50	56	21	77	37	19
51–100	17	8	25	11	6
> 100	17	5	22	12	5

STEC: Shiga toxin-producing *Escherichia coli*.

^a^ Food and/or environmental samples available for testing as part of the outbreak investigation.

^b^ Food and/or environmental samples not available for testing as part of the outbreak investigation.

^c^ Outbreak strain successfully isolated from food and/or environmental samples during the outbreak investigation. Only outbreaks where samples were available are included in the count.

^d^ Outbreak strain not isolated from samples taken from implicated food and/or environment during the outbreak investigation. Only outbreaks where samples were available are included in the count.

When we analysed the outbreaks by attributed food vehicle category (either by microbiological testing and/or epidemiological evidence), ‘beef’, ‘salads and leafy greens’ and ‘unpasteurised dairy products’ accounted for 62% of all outbreaks. *Escherichia coli* O157 was the predominant serotype for each food category except for ‘pasteurised dairy products’, however, this represents a very small sample size (n = 5 with *E. coli* O157 associated with two outbreaks, non-O157 STEC associated with two outbreaks and one outbreak due to dual infection).

We compared outbreak size and attributed food vehicle category (Table 2). Outbreaks attributed to beef were distributed across all outbreak size categories, while unpasteurised dairy products were more commonly implicated in smaller outbreaks, and salads and leafy greens were not implicated in any outbreaks involving fewer than five cases.

**Table 2:** Outbreak size and food vehicle category implicated in food-borne outbreaks involving STEC (n = 223 outbreaks)

Food vehicle category	Number of cases in outbreak					Total
	< 5	5–15	16–50	51–100	> 100	
	n	n	n	n	n	
Beef	9	27	22	7	5	70
Salads and leafy greens	0	10	13	9	6	38
Unpasteurised dairy products	8	12	9	1	1	31
Other	5	28	33	8	10	84

STEC: Shiga toxin-producing *Escherichia coli*.

For outbreaks where samples were available for testing (n = 137) we reviewed the isolation outcome for each food category (Table 3). The data suggest a reduced chance of successful isolation from salads and leafy greens, where only half of these outbreak investigations achieved successful isolation of the outbreak strain from food and/or environmental samples. This was compared with beef, which had the highest number of outbreaks with samples available for testing. A generalised linear model showed that the outbreak strain is significantly less likely to be isolated from salads and leafy greens than from beef (odds ratio = 0.16; 95% confidence interval: 0.04–0.54; p = 0.004).

**Table 3:** Number of reports on food-borne STEC outbreaks and isolation status by food category (n = 137 outbreaks)

Food category	Failed isolation of outbreak strain		Successful isolation of outbreak strain		Total number of outbreaks^a^
	n	%	n	%	n
Beef	6	14	38	86	44
Unpasteurised dairy products	8	31	18	69	26
Salads and leafy greens	9	50	9	50	18
Other meat	4	27	11	73	15
Bakery products (contaminated flour)	2	40	3	60	5
Pasteurised dairy products	3	60	2	40	5
Food categories with < 5 studies or where a single causative food item/ingredient could not be identified	11	N/A	13	N/A	24
**Total**	**43 **		**94 **		**137 **

N/A: not applicable; STEC: Shiga toxin-producing *Escherichia coli*.

^a^ Outbreaks where no samples were available for testing were removed from the analysis.

Of the 137 outbreaks with samples available for testing, a genetically distinct isolate or an additional strain to the outbreak strain was identified in 27 (20%) of outbreaks. For 18 of these outbreaks, the outbreak strain was detected alongside one or more additional strains, and for the remaining nine the outbreak strain was not isolated, but a different STEC strain was.

Isolation of the outbreak strain was unsuccessful in 129 (58%) of the total outbreaks reviewed in this study. For the majority of these outbreaks (n = 89; 69%), the report(s) documented that the failure to isolate the outbreak strain was because there were no samples available for testing. For the remaining 40 outbreak investigations (31%), we categorised the reason(s) given by the author(s) as to why the outbreak strain was not isolated in Table 4. Where more than one potential reason for the failure to isolate the outbreak strain was given, the primary reason was included in this analysis. Where the categorisation is “unknown - not enough information to specify” this reflects either the authors stating that there was not enough information to conclude why this outbreak strain was not isolated, or that this information was not provided in the outbreak investigation report(s).

**Table 4:** Reason given where the STEC outbreak strain was not isolated from food or environmental samples in outbreak investigations (n = 129 outbreaks)

Reason for not isolating the outbreak strain	n	%
No food or environmental samples available for testing	89	69
Implicated batch not available for testing (alternative batch tested)	7	5
Inconclusive epidemiological/ trace-back investigation therefore sampling potentially not directed to correct food item/premises	10	8
Sampling methodology inadequate	2	2
Isolation failed due to food vehicle attributes	4	3
Failed due to laboratory method limitations/low level contamination	5	4
Unknown - not enough information to specify	12	9
**Total**	**129**	100

STEC: Shiga toxin-producing *Escherichia coli*.

From the 129 outbreaks where the outbreak strain was not isolated from food or environmental samples, 37 specified the risk management action taken for the incident. In 16 of these 37 incidents, the risk management decision was to take no action. For 10 outbreaks, no rationale for this risk management decision was provided, and the reasons given in the remaining six were: that the outbreak was over before action could be taken (n = 4), that the investigation was inconclusive (n = 1) or that a home-made product was implicated (n = 1). In the remaining 21, some form of risk management action was taken based on epidemiological evidence alone: product recall from consumers (n = 15), recall and wider control measures (e.g. import controls) (n = 2), recall and enforcement action (i.e. restriction on production at premises) (n = 1), a withdrawal from the market (n = 2), and enforcement action (i.e. suspension of activity at premises, requirement for pasteurisation of milk for dairy product production) (n = 1).

We assigned critical appraisal scores to each publication, or the main publication where more than one publication reported on the same outbreak. A total of 103 reports (46%) were assigned a score of 1–2 and 120 (54%) were assigned a score of 3–5. All relevant reports were included in the analysis irrespective of critical appraisal score. We compared the association between peer-reviewed literature and grey literature sources with the chance of success of isolation, and found that outbreaks where the outbreak strain was successfully isolated from food or environmental samples were twice as likely to be reported in peer-reviewed sources (χ^2^ = 10.624; df = 1; p < 0.001).

## Discussion

Most STEC outbreaks identified were caused by *E. coli* O157. Although the wider introduction of PCR-based methods has improved detection of non-O157 STEC, the testing algorithms used by clinical laboratories during the time frame of this study focused on the use of culture media (cefixime, tellurite sorbitol MacConkey (CT-SMAC) agar) selective for STEC O157. Historically, STEC O157 was considered to be a more common cause of severe symptoms than other STEC serotypes, although there is recent evidence that adverse clinical outcomes are frequently caused by non-O157 STEC [11,12]. Although the use of CT-SMAC medium has the potential to facilitate the detection of STEC O157 over non-O157 STEC, we identified no significant difference in isolation success rate from food/environmental samples between outbreaks attributed to either group. 

In the majority of outbreaks where an STEC was found, it matched the clinical cases. However, in a fifth of outbreaks, an STEC strain genetically distinct from the outbreak strain was detected and in a third of these, only a genetically distinct STEC strain was detected in the implicated food. The presence of different STEC strains demonstrates that the food safety management systems in place are not sufficient to prevent STEC contamination of the final product.

Although larger outbreaks were more likely to have samples taken, we found no statistically significant association between the likelihood of outbreak strain isolation and the size of the outbreak. This was an unexpected finding as outbreaks with more cases will often have more patient information, more food for sampling and potentially more resources for epidemiological investigations. The reasons for this lack of association are likely to vary, and are discussed in further detail below, however, the most important is probably lack of available food for testing, e.g. due to short shelf life or rapid product turnover which may affect both large and small outbreaks. Therefore, environmental sampling should be considered early in outbreak investigation, for example at primary production sites such as farms. The resources to investigate an outbreak will vary greatly and will need to be directed on the most likely source. It is unlikely that there would be resources to undertake untargeted sampling, making it harder to identify novel or unexpected sources.

Hospitalisation data were not available for all outbreaks and have not been included in this analysis, but where such data were provided, we included them in the Supplement. For some outbreaks, the report(s) provided total HUS cases and deaths, but as this was reported inconsistently it was not included in the analysis.

The number of outbreaks where a specific food vehicle was not identifiable from epidemiological evidence was similar across the five outbreak size categories. There was however an association between outbreak size and food type. Unpasteurised dairy products were more commonly associated with smaller outbreaks and were implicated in 36% of outbreaks involving fewer than five cases, but only 5% of outbreaks of 50–100 cases or more than 100. Conversely, there were no outbreaks of fewer than five cases attributed to salads and leafy greens, although this food was implicated in 63% of outbreaks involving 50 cases or more. This may reflect the structure of the food chain (for example unpasteurised dairy may be more likely to have local, small-scale distribution, or be more readily recalled in food histories), or emphasise that salads and leafy greens are particularly difficult to identify due to their perishability and limited recall in food histories by patients. It is unlikely that salads and leafy greens did not cause any small outbreaks, suggesting under-identification of small outbreaks caused by them.

In line with other studies such as [13], we found that the most common food types associated with STEC outbreaks were beef, salads and leafy greens and unpasteurised dairy. We also identified outbreaks attributed to a range of sources beyond these top three food types, with 16 outbreaks representing eight categories of food including sprouts, vegetables, herbs, nuts/nut butters, beverages, fruit and fruit juices, pickled or fermented vegetables and ready-to-eat prepared dishes and dips (e.g. coleslaw, guacamole).

The report authors did not routinely indicate why they believed their investigations had been successful in isolating the outbreak strain from food or environmental samples. This meant it was not possible to provide any analysis of factors in an investigation which contributed to successful isolation of the causal strain, and therefore why the focus of this analysis is on the reasons given for the outbreak strain not being successfully isolated. The converse of the reasons for unsuccessful isolation are likely to hold though, such as established testing methods for specific well-known food matrices.

For most of the outbreaks included in this study where the isolation outcome was unsuccessful, it was attributed due to a lack of sample availability (89 outbreaks).

For seven outbreaks included in the study, an alternative batch was tested and found to be negative for the outbreak strain. Although persistent contamination on a farm, or in a production setting may also lead to contamination in subsequent batches, non-homogenous distribution through food matrices, differing conditions between batches, or intermediate contamination of batches make it unsurprising that the outbreak strain was not detected in alternative batches. For example, a systematic review of STEC outbreaks linked to sprouted seeds and salads found six studies with strong evidence for bacterial contamination having been introduced from irrigation water or animal faeces [14]. In these cases, the exposure to contaminated irrigation water may be unlikely to persist between batches, particularly where contamination had arisen from flooding events.

We categorised 10 outbreaks as ‘inconclusive epidemiological/trace-back investigation’, i.e. the sampling was unlikely to be directed to the correct food item or premises or was being used to investigate multiple possible sources. These were mainly outbreaks where a complex restaurant supply chain or buffet were involved, or an ingredient which was difficult to recall in food histories, such as a garnish, was implicated [15-18]. Certain food products, for example, microgreens or herbs and spices, can be referred to as ‘stealth vehicles’ because they may be consumed without realising, as additions to meals when eating out of the home [19]. It is important in incident investigations to consider garnishes and ingredients which may be used by restaurants in multiple dishes as a factor that can complicate attempts to identify the food vehicle. An author reported using photos to help cases distinguish which sprouts they consumed in a buffet setting [20]. In addition, the distribution chain for salad products is often complex, with multiple farms supplying individual retailers [21,22] or cases arising in multiple countries or regions [23,24]. This may make traceback to specific farms complicated.

For outbreaks categorised as ‘inadequate sampling methodologies’, ‘attributes of the food vehicle involved’ or ‘laboratory method limitations/low level contamination’, we acknowledge that although the categorisation was based on the way the authors described the isolation outcome, there is the possibility of these categories overlapping. In all three categories, the difficulty of detecting low-level or heterogeneously distributed pathogens was clear. For example, all four outbreaks related to alfalfa sprouts had samples available for testing. The heterogeneous nature of contamination, with only a small percentage of seeds likely to be affected was the reported explanation why the outbreak strain was only detected for one of these outbreaks [25]. This reflects known difficulties in isolating STEC from certain foods or food matrices, and the specialist techniques required to successfully isolate from these matrices [26]; for example blue cheese is known to be a complex food matrix due to distinct microenvironments between the core, rind and veins [27]. Detection of low levels of contamination may be difficult, even at a level that may make some consumers unwell. In an outbreak of *E. coli* O157 related to raw milk cheese in Canada, the authors reported that the 60-day ageing process to lower bacterial counts reduced the capacity for detection, while still allowing enough bacterial survival to infect some cases [28]. Similarly, enhanced testing was required to detect STEC in watercress irrigation water [29]. The difficulty in identifying low levels of contamination can be amplified by limited quantities of product from a batch being available for testing [25,30].

Although outbreak investigation involves three pillars (epidemiology, microbiology and food chain investigations), robust associations between particular food types and clinical illness can be established using epidemiological data alone [9] for example through case–control or case–case studies. As sequencing becomes more commonly used, databases will be built up that allow identification of an implicated food item before any epidemiological analysis has been carried out by linking a human isolate to a food isolate. 

The literature search for this study was conducted in September 2019, therefore although the cut-off for inclusion was outbreaks reported up to August 2019, it is likely that outbreaks at the upper end of the time range will have been missed as they had not yet been published. Lack of resources, in-part due to the COVID-19 pandemic, led to a delay in publishing this research. We detailed in the methodology section the range of resources that were consulted to identify outbreaks, we acknowledge that there may have been additional resources which we were not aware of at the time of the review such as the CDC NORs resource. 

Although 46% of outbreak reports were given a critical appraisal score of 1–2, this does not mean that we had concerns about the quality of the publication, rather that these reports detailed only one or two of the aspects of a comprehensive outbreak investigation. We included all relevant outbreaks in the analysis irrespective of the report critical appraisal score, with the aim of overcoming publication bias that can result in published literature disproportionately reporting on large or novel outbreaks. Although we tried to address this bias by including all outbreaks identified, we recognise that there is still likely to be under-reporting of incidents where the food vehicle was not identified. It is also important to note that the food-borne outbreak investigation systems are not globally harmonised, therefore the differences in the number and type of reported outbreaks, as well as in the causative agents, may not necessarily reflect the level of food safety; rather, they may indicate differences in the systems used to identify and investigate food-borne outbreaks [31]. We note that the availability of resources to conduct sampling can vary by country or even by year. 

Additional limitations of this study include: the consideration of reports published in English only, the challenge of designing a search strategy to capture all relevant outbreaks, differences in information reported by different authors and the relatively small number of reports identified for inclusion in the study. Finally, we acknowledge that the definition of an outbreak has changed over the time period investigated as advances allowed more accurate determination of clusters. For example, by whole genome sequencing, a cluster would typically be expected to be within five single nucleotide polymorphisms (SNPs), whereas previous methods may have linked cases by serotype. Not enough detail was available in the outbreak descriptions reviewed here to analyse any effect this may have had on the findings of this report. For many of the investigations included in this study, only limited information was provided on the risk management decisions taken, and information on the reasons underlying the decision making was not routinely provided.

## Conclusion

We have used STEC as a model pathogen to investigate the reasons why an outbreak strain may not be identified in an implicated food. While it is not essential to isolate the outbreak strain to identify the cause of an outbreak and take action, finding it does provide further evidence to support a hypothesis. Over half (58%) of the outbreak investigations included in this review did not isolate the STEC strain. The reasons for this include failure to acquire food or environmental samples, and properties of the food product which can make detection, particularly at low levels, difficult. We recommend that investigators take steps to increase the likelihood of obtaining microbiological evidence from implicated foods. These include routinely including questions on garnishes and other ’stealth vehicles’ when gathering food histories, contacting restaurants where cases have eaten to detect any signals quickly, sampling perishable products such as salad leaves rapidly to ensure the opportunity is not missed (although it is recognised that the shelf life for a batch may be as short as the incubation period for STEC symptoms to develop), considering on-farm environmental sampling, and developing generic structured food sampling plans before outbreaks occur that can be adapted and initiated at pace.

## Supplementary Data

113000 Supplement
